# Pulse Oximetry Screening in the Newborn: Can Modifications to the Screening Algorithm Improve Detection?

**DOI:** 10.3390/ijns12030053

**Published:** 2026-07-20

**Authors:** Andrew K. Ewer

**Affiliations:** College of Medicine and Health, University of Birmingham, Birmingham B15 2TT, UK; a.k.ewer@bham.ac.uk

**Keywords:** pulse oximetry screening, critical congenital heart defects, newborn babies

## Abstract

Pulse oximetry screening (POS) is a simple non-invasive tool which enables the early detection of critical congenital heart defects (CCHDs). POS has moderate sensitivity and high specificity; it is cost-effective, readily accepted by parents and healthcare professionals, and its introduction significantly reduces mortality from CCHDs. As a result, most high-income countries and many middle-income countries have introduced, or are considering introducing, POS. Despite the international uptake of POS, there is still no consensus regarding the most appropriate screening algorithm, although several have been described. In addition, this review will consider the variations—and their relative advantages and disadvantages—between POS algorithms and also explore how modifications to existing algorithms—such as the addition of a perfusion index or the incorporation of machine learning—may have the potential to improve future detection of CCHDs.

## 1. Background

Congenital heart defects (CHDs) are the most common congenital abnormality with an incidence of 8–10 per thousand live births [1]. CHDs are an important public health concern accounting for 10% of all infant deaths and 46% of deaths related to congenital malformations [2,3,4]. Nearly a quarter of all CHDs are defined as critical (CCHDs), i.e., requiring surgery or catheter-based intervention in the first month of life [4]. The majority of CCHDs are treatable particularly if diagnosed in a timely manner before acute deterioration. Newborn screening based on pre-discharge physical examination alone misses up to 50% of CCHDs [4]. Approximately 40% of these neonates present with cardiogenic shock, and in 5% of them diagnosis would only be made at autopsy [5,6]. Most neonates with CCHDs remain asymptomatic while the ductus arteriosus remains patent, providing an opportunity for screening in the first day or so of life [4]. The timing of diagnosis has an impact on outcome, and neonates diagnosed before postnatal collapse have better long-term survival and neurodevelopmental outcomes [7,8,9].

Prenatal diagnosis of CCHDs allows for perinatal optimisation, selection of place of delivery and timely intervention. However, despite advances, there remains a significant variation in the antenatal detection rate of CCHDs, both between different countries and also between institutions within the same country, with an average detection rate of about 50% [10,11,12,13]. This variation may be attributed to disparities in healthcare resources, screening programmes, and individual operator expertise [14].

Although hypoxaemia is present in the majority of CCHDs, this may not always be clinically apparent [15]. In addition, cyanosis may be difficult to detect in neonates with darker skin tones [11]. The rationale for POS is to detect the subclinical hypoxaemia associated with CCHDs in otherwise asymptomatic neonates.

A comprehensive Cochrane systematic review of 21 studies with over 457,000 participants showed that the sensitivity of pulse oximetry for detection of CCHDs was 76%, while specificity was 99.9% with a false-positive rate of 0.14% [16].

POS has been shown to be cost-effective [4,17], acceptable to parents and clinicians [4,17], and it can reduce mortality from CCHDs by over 30% [18]. However, POS is not a perfect test and despite best efforts, some CCHDs (particularly left heart obstructive lesions such as coarctation of the aorta) are detected less frequently than others [19] and as such, a small minority of babies with CCHDs remain undiagnosed at discharge despite undergoing comprehensive routine screening.

## 2. Variation in Screening Algorithms for POS

Despite general agreement among clinicians that POS is beneficial in identifying babies with CCHDs before discharge from hospital, there are several variations in the screening process, particularly relating to the screening algorithm used [19].

The main reported differences include: (i) use of both pre- and post-ductal saturations (right hand and either foot) versus single post-ductal measurement (foot only)—the former also takes into account differences between the two measurements, even when both are within normal limits and (ii) timing of screening (i.e., before or after 24 h of age).

In algorithms using both pre- and post-ductal measurements there are also differences in the inclusion of low saturations (<95%) in one or both limbs, the absolute value of the differential measurement between the two in determining a positive result and the number of retests following a borderline result [19].

When evaluating the test accuracy of specific algorithms, it is important to consider a number of factors, including sensitivity, specificity, and false-positive (FP) rate. It is also vital to ensure that testing results in a timely diagnosis—before presentation with acute cardiovascular collapse [19]. As stated above, the Cochrane meta-analysis of all POS studies confirmed modest sensitivity and high specificity; however, it did not identify any significant difference in sensitivity between pre/post vs. post-ductal testing or timing of the test [16].

As a result of these uncertainties, national POS algorithms vary between countries. Several, including Germany, Austria, Ireland and New Zealand, have recommended post-ductal-only screening [20,21,22,23], perhaps because of its relative simplicity (one measurement only), concerns about workload for screeners and the possibility of miscalculating the result. Increasingly, however, the majority of national recommendations include dual-site screening [20].

Later screening (after 24 h) is recommended in the USA [24], Canada [25], Germany [21] and Austria [22], whereas early screening (before 24 h) is recommended in the UK [26], Nordic countries [27], Latin America [28], Saudi Arabia [29], Spain [30] and Sri Lanka [31]. A European consensus statement also recommended screening between 6 and 24 h of life using both pre- and post-ductal measurements [32]. The question remains: do these variations make a difference to detection rates?

Despite the findings of the Cochrane meta-analysis [16], it is still useful to investigate the POS results in individual babies, particularly those with CCHDs which were missed. Although the evidence is somewhat limited, when raw saturation data from babies who had pre- and post-ductal measurements are analysed, it is clear that some babies with CCHDs would be missed by post-ductal testing alone [19]. For instance, in coarctation of the aorta, a difference between pre- and post-ductal saturations often occurs, even when both are normal. In transposition of the great arteries, particularly with associated aortic obstruction, post-ductal saturation may be normal while pre-ductal saturation is low (reversed cyanosis) [19,33].

**Figure 1:**
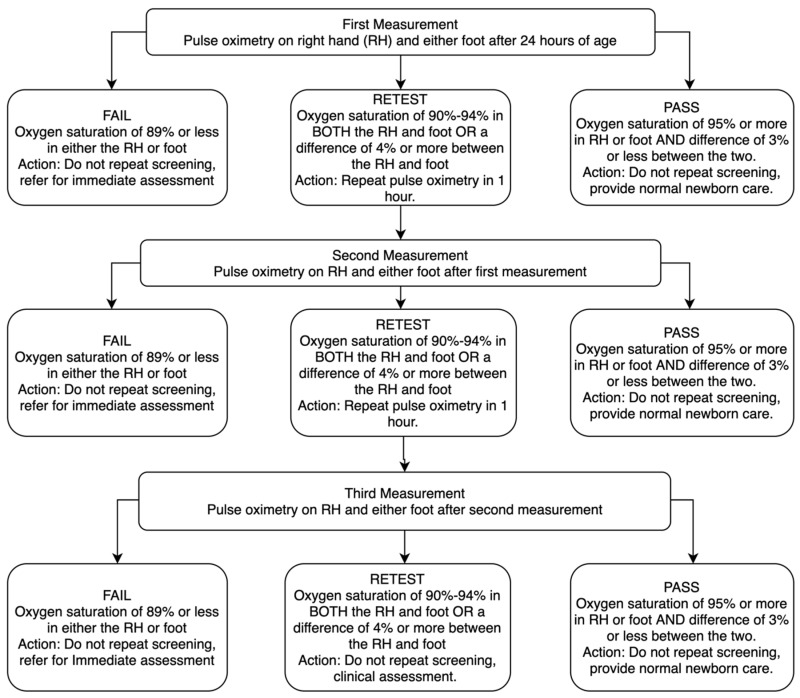
Original USA algorithm for POS.

Meta-analyses have consistently shown that the FP rate in studies that screened later (after 24 h of age) is much lower than in those that screened early (within 24 h) [16,34]. As some healthy newborn infants will have transitional circulation in the first few hours which may result in mild transient hypoxaemia, this is perhaps not surprising. This increase in the FP rate has led to many countries (including the USA) adopting a later screening algorithm [24] (Figure 1). Thus, while later screening results in fewer FP results (FP rate—0.47% < 24 h vs. 0.11% > 24 h) [16], there is good evidence from later screening studies [35,36] that up to 50% of the infants with CCHDs present with symptoms in the first day of life, and around 10% will have pre-diagnosis postnatal collapse [35], which is the very event that screening aims to prevent. Additionally, early screening is also more likely to detect potentially life-threatening non-cardiac conditions that require early intervention [37,38,39]. These factors must be considered carefully; although low FPs are important in a screening test, if the majority have a serious non-cardiac condition which requires urgent treatment, this is clearly a significant additional clinical benefit [16,38,39]. Additionally, postnatal care practices also vary between different countries. In many countries, most otherwise-healthy infants are discharged soon after birth, and there are increasing numbers of home births where the midwife leaves the baby a few hours after birth—both practices make later screening challenging [40,41].

In the USA, the POS algorithm recommended by the American Academy of Pediatrics (AAP) in 2011 [24] was described in a large Swedish trial [35] where screening took place after 24 h, measuring pre- and post-ductal oxygen saturations (Figure 1); it reported a low FP rate of 0.17%. The screening is passed if either saturation result was >94% or the difference between the two was <4%. A positive screening meant that saturation measurement was <90%, and a retest was advised if both saturations were between 90 and 95% and/or the difference between the two was 4% or more. The algorithm allowed for two more retests at 1 h intervals before the test was deemed positive, and referral for further assessment was necessary. This algorithm has also been recommended in Canada [25].

Interestingly, the authors of the Swedish study led by Anne Granelli highlighted that a significant proportion of babies with CCHDs presented before screening and therefore suggested that screening in the first 24 h was likely to be preferable [35].

**Figure 2:**
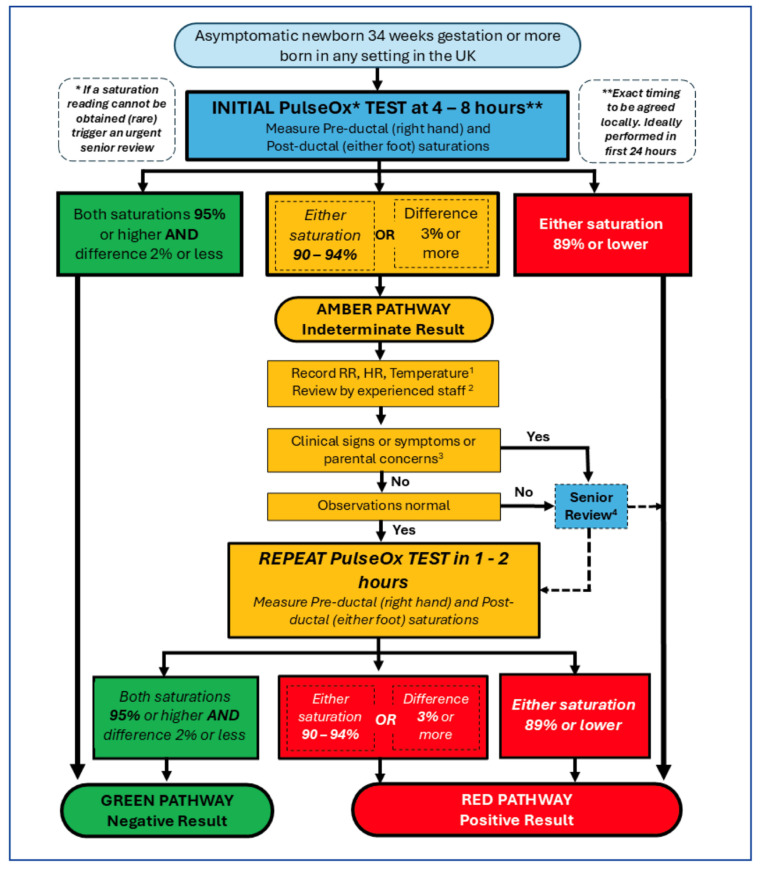
Protocol for the PulseOx test, adapted from the BAPM framework: routine pulse oximetry testing for newborn babies. (Courtesy of V Monnelly, MRCP, Edinburgh UK).

In the UK, the majority of screening units [42] have adopted the algorithm described in the UK PulseOx study published in 2011 [37], where babies were screened in the first 24 h using pre- and post-ductal saturations. The definition of a positive test is slightly different from the original USA algorithm (Figure 2) [24,26,37]. With this approach, a positive test is recorded if *either* saturation result is <95% and/or with a difference of >2%. Any positive test is followed by a clinical assessment. If the clinical assessment is abnormal, more detailed assessment is advised. With a normal clinical assessment, a single repeat screen is performed after 1–2 h. Using this algorithm in the original study and in subsequent clinical practice, the FP rate was 0.8% [37,38]. However, 79% of the FP cases had significant medical condition [38]. Less than one-third of infants who had a positive test underwent echocardiography, mainly because an alternative non-cardiac diagnosis had been identified [38].

Further evidence from the UK suggests that the performance of the PulseOx algorithm is maintained despite an increase in antenatally detected CCHDs [39]. Over a six-year period, the proportion of healthy babies admitted to the neonatal unit following a positive test fell from 29% to 2.4%, largely as a result of staff confidence in assessing borderline test-positive babies in the well-baby nursery [39].

Recently, the British Association of Perinatal Medicine (BAPM) has produced a framework for practice for implementation of POS for all neonates born at >34 weeks’ gestation, utilising the PulseOx algorithm (Figure 2) [26].

**Figure 3:**
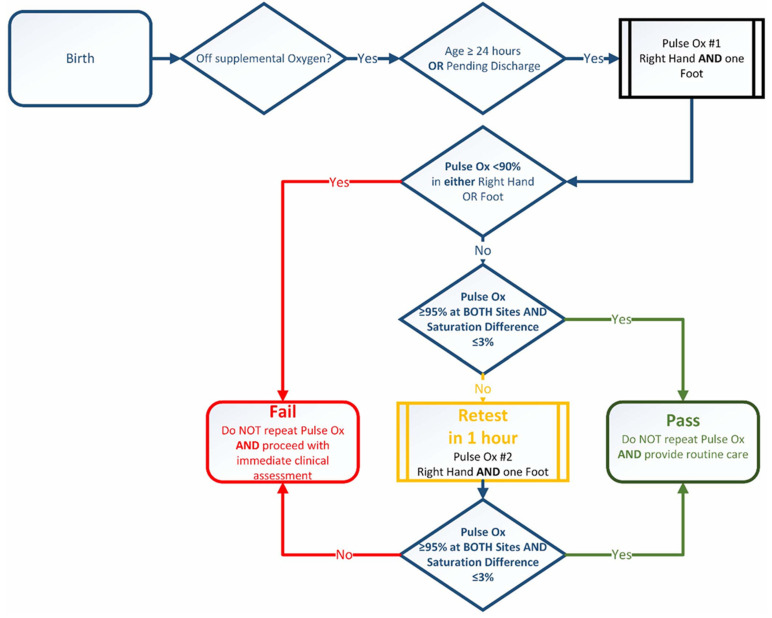
Recommended updated algorithm by the AAP for the newborn screening of critical congenital heart defects using pulse oximetry. Reproduced with permission from *Pediatrics*, 155 (1), e2024069667, [44], Copyright © 2025 by the AAP.

In the USA in 2018, an expert workgroup convened a meeting to discuss modifications to the USA algorithm and recommended that *both* pre- and post-ductal oxygen saturation should be >95% for a ‘pass’ result and to reduce the repeat screen to only one retest in the case of an inconclusive test result [43]. Although earlier screening was not recommended, the workgroup agreed that it was acceptable. Very recently, the AAP updated their recommendations on the use of POS in the USA in line with the changes suggested by the workgroup (Figure 3) [44], with the aim of reducing confusion and misinterpretation, reducing the time to recognise CCHDs and potentially increasing the sensitivity without significantly affecting retesting rates. These changes make the USA algorithm more aligned with the UK version [26,37]; however, the recommended time of testing has not changed.

## 3. Improving Detection of Left-Sided Obstructive Defects

The addition of POS to existing screening methods ensures that over 90% of newborns with CCHDS are identified before discharge home, compared to the 50–70% detection rate with newborn exams alone [4]. However, the sensitivity of POS for detecting left heart obstructive defects (LHODs) such as critical coarctation of aorta, interrupted aortic arch and critical aortic stenosis remains low and is reported by some studies to be below 50% [19]. Unfortunately, the presence of the ductus arteriosus means these defects are also the most difficult to identify by antenatal ultrasound and early clinical assessment. A patent ductus also means that saturations may be within normal limits with these defects, making it more difficult to detect by POS. A more conservative cut-off for the pre/post difference (such as the PulseOx algorithm) [26,37] may increase the detection of these defects, but there is currently insufficient data to support this [19]. More national data from babies with CCHDs screened with POS may help with this, but these have not yet been made available. To overcome this limitation, researchers have attempted to incorporate other data which can be obtained from routine POS into the screening algorithm to enhance detection of LHODs.

For a number of years there has been considerable interest in the use of perfusion index (PI) as a potential additional screen for LHODs. PI is an objective assessment of the peripheral pulse which indicates the ratio of pulsatile blood flow to non-pulsatile or static blood flow in peripheral tissue. PI can be measured non-invasively by new generation pulse oximeters at the same time as oxygen saturations and used as an indirect measure of peripheral perfusion [45,46]. As peripheral perfusion is often compromised in aortic obstruction, the potential usefulness of PI as a screen for these defects appears logical.

Several studies have attempted to investigate two important parameters—(i) the normal range of PI in healthy newborn babies and (ii) the threshold at which PI may become useful for detecting CCHDs, particularly LHODs—both of which are essential prerequisites for any potential screening test.

A Swedish study first evaluated the addition of PI to POS as a means of increasing test accuracy for LHODs in a multicentre, prospective study involving 10,000 healthy newborns [47]. The study reported a median post-ductal PI of 1.7 (IQR of 1.2 to 2.5) at 1–120 h of life [47]. Using the 5th centile for PI in this group (0.7) as a threshold cut-off level for normal perfusion, they then tested nine neonates with previously diagnosed duct-dependent systemic circulation (LHODs) and a PI < 0.7 (pre- or post-ductal) was observed in five cases, (sensitivity—56%) including two babies (one CoA, one HLHS) who had normal newborn examination and POS. Although providing interesting data, this study was not without limitations, particularly when evaluating the use of PI as a screening test. The babies with CCHDs were already diagnosed; some were on prostaglandin treatment and were therefore perhaps a group with the potential for bias. Also, PI was measured after 36 h of age in 56%, which reduces the applicability of the data, particularly for POS screening algorithms which recommend screening around the first 24 h of life.

In a further study incorporating data from 2768 POS results, the median PI in asymptomatic newborns at 24 h of age was 1.8, (IQR 1.2 to 2.7) [48], closely aligning with the results from the Swedish study, suggesting a consistent and narrow range of PI values among asymptomatic newborns during the period where POS usually takes place.

In a large, multicentre, prospective study involving over 42,000 apparently healthy newborns, a team from Italy evaluated the addition of PI to POS after routine physical examination [49]. The cohort were screened using PI with a higher PI cut-off value (0.9) and later in postnatal life (between 48 and 72 h of age). Unfortunately, the prevalence of CCHDs in the screened population was low (as a result of a high antenatal detection rate of 76%), and 84% of the remainder were diagnosed before screening took place. Only seven babies with CCHDs (out of a total cohort of 187) were screened and four were missed by both POS and PI (including two CoAs and one IAA). PI picked up one CoA that was missed by POS and the two remaining CCHDs were picked up by POS alone.

In a small, single-centre, prospective study involving just over 3000 apparently healthy newborns, Uygur et al. also recorded PI at the time of POS [50]. Thirty-three neonates with antenatal diagnoses of CCHDs were included, and much higher cut-off values for PI were used (1.2 for pre-ductal and 1.2 for post-ductal results). In the group without antenatal diagnoses, 10 newborns were diagnosed with CCHDs: seven due to positive POS alone and three due to heart murmur with negative pulse oximetry (one baby with CoA also had low PI). In the group with antenatal diagnosis, five neonates with confirmed CCHDs had normal POS, including two with low PI. One neonate with LHODs had normal POS with low PI values but also had abnormal physical examination findings that led to the diagnosis. FP rates were high—2.7% for pre-ductal and 3.6% for post ductal. When the more usual cut-off of 0.7 was applied, sensitivity fell from around 60% to just over 30%. The authors concluded that there was no additional benefit for PI over standard screening methods combining antenatal ultrasounds, POS and physical examination.

A single-centre, prospective study involving over 1000 newborns evaluated the CCHD-detection rate using combined PI (cut-off of 0.7) and POS between 24 and 72 hours old [51]. Four CCHDs were identified due to positive POS, with only one of these also having low PI (and a pathological murmur). The study concluded that PI screening offered no additional benefit for over-saturations and clinical examination.

In a small study, Siefkes et al. measured PI after 24 h of life (at the same time as POS) in 123 healthy newborns and reported the 5th centile for post-ductal PI to be 0.5 [52]. When they applied this threshold value to 13 babies with known CCHDs (including four with LHODs), they showed that PI would pick up three out of four (75%) of the CoA/IAA defects, all of which had been missed by POS. However, 2.44% of normal infants had false-positive PI screenings.

In summary, these studies show that using PI as an additional screening test can identify some babies with LHODs that would have been missed by POS (and other screening tests). This is of interest and potentially important. However, detailed exploration makes the case for PI screening less clear-cut [46,53]. Most studies were underpowered. The identified ‘normal’ values for PI vary significantly between studies, as do the screening thresholds used, possibly influenced by the time of screening, but this does not explain the differences completely. In addition, most screening studies report a single PI measurement, and very few report longitudinal measurements within an individual baby, which might demonstrate both the stability of PI over time and how representative one measurement is as a reflection of actual perfusion [46,53].

Studies showed which showed moderate sensitivity for PI used cohorts where the diagnosis of LHODs had already been established, and many were receiving prostaglandin treatment, thus representing a pre-selected group rather than an apparently healthy population that would usually undergo screening. There is a risk of bias when PI measurements take place unblinded at a later time after birth than POS would take place and where the pathological circulation has been modified [46,53].

A very recent study from another Swedish group addresses some of these issues. Lannering et al. identified normal (control) PI data from a cohort of 512 apparently healthy term newborns measured concurrently with routine newborn POS [54]. They then compared these PI results with those from 38 cases of newborn babies with proven LHODs (36 cases of CoA and two cases of IAA) from across Sweden who had also had PI recorded with routine POS over a 6-year period. Although there is no recommendation for PI screening in Sweden, 13/46 (28%) of Swedish maternity units record, and act on, PI data acquired at the time of POS, although different thresholds are used for a positive result.

Only three control infants had a PI of <0.7 on three consecutive occasions (which was the threshold for 11/13 of the units) but subsequently underwent normal echocardiography (FP rate 0.6%). POS was normal in all controls.

Data from those babies who had LHODs revealed some important findings. Firstly, none of the infants with LHODs were diagnosed as a result of a positive PI screen—although three of these infants had a PI of <0.7 at some point, they improved in two, and for one the local threshold was <0.5%. Although four infants were picked up by POS and 14 by newborn examination, the majority, 21/38 (55%), were discharged without a diagnosis, and well over half of these (57%) presented with acute circulatory compromise. However, further analysis of the PI data from the critical AAO group identified previously undescribed new findings. The median pre-ductal PI value in the LHOD group was significantly higher compared with the median post-ductal values and the pre-ductal values of the control group (*p* < 0.001 for both). Using these data, the team then tried to identify the optimal threshold value to discriminate between controls and LHODs and found that a PI of >3% in the right hand had the highest sensitivity (57.9%). This was higher than using either POS and/or newborn examination results, but specificity was 86.1% and the false-positive rate was 13.9%. Combining POS, examination and right-hand PI results gave a higher sensitivity of 76.3% but with a lower specificity (85%) and higher false-positive rate (15%). If right hand PI > 3% had been included in the screening criteria, then 12 more critical AAO defects would have been detected and prevented from being discharged undiagnosed, compared to using only POS and NPE, thus potentially reducing the number of critical AAO defect discharges by almost 60%.

These novel findings are reported from the largest published LHOD cohort to date, which importantly includes both POS and PI data before the diagnosis was suspected or treated. In addition, most babies were screened within the first 24 h (median 10 and 18 h for controls) so the possibility of babies presenting before screening could take place was reduced [55]. Unlike many other reports, the definition of a critical CCHD is robust (death or treatment within 28 days) and consistent with the reference literature [16]. However, the control group is very small, and more data from much larger normal populations are required to corroborate these findings.

The very low sensitivity rate for detection of LHODs (7.9%) using the frequently quoted PI threshold of <0.7% in such a large robust cohort strongly suggests that this is unlikely to add value to routine screening. However, the PI threshold of >3% in the right hand offers a greater opportunity if this can be confirmed in larger populations. Unfortunately, as with most studies investigating the added value of PI in the detection of CCHDs, the false-positive rate remains unacceptably high [55].

Although there is some evidence to suggest that the addition of PI to POS may identify infants with LHODs who would have otherwise gone undiagnosed with current screening methods, this is currently insufficient to suggest PI would make a robust screening test.

## 4. Machine Learning-Based Critical Congenital Heart Defect Screening

Machine learning (ML) uses algorithms to analyse and interpret continuous data, filter out noise, recognise relevant patterns, and make predictions of true events. Recent attempts have been made to incorporate data, in addition to oxygen saturations, gathered from photoplethysmography, including PI, diastolic and systolic waveform components and radio-femoral delay, to aid detection of CCHDs using ML.

In a proof-of-concept study Lai et al. enrolled 335 newborns and collected routine data, including heart rate (HR), perfusion index (PI), and oxygen saturations between birth and >48 h, dividing the data into two groups: 0–48 h (158 healthy, 27 CCHD) and over 48 h (50 healthy, 36 CCHD) [56]. Compared to POS alone, the proposed enhanced system, which integrated the trained ML models, demonstrated a sensitivity improvement of approximately 10% with no additional false positives when used over 48 h. When used between 0 and 48 h, addition of the ML model resulted in three false positives and detection of three additional newborns with CCHDs [56].

In a further study, the same group enrolled 523 newborns, including 132 with CCHDs, among whom 21 had isolated CoA. They compared four ML models with measurement at one or two time points and with or without incorporation of radio-femoral delay. Unfortunately, the final analysis was limited to 79 patients (14 with CCHDs and only three with CoA). Although underpowered, the study demonstrated that the best-performing model included two time points and a pulse delay, which improved CCHD detection from 71% (with POS alone) to 93%, although neither this difference nor the difference in the rate of detection of CoA was statistically significant [57].

While these studies are interesting and show promise, the use of ML in healthcare is still at a nascent stage. Further prospective studies that include larger numbers of patients are required to establish the generalisability and reliability of these results. An ideal ML model would use the data from existing POS protocols to reliably improve the detection of left-sided obstructive lesions without an increase in false-positive results, thus ensuring no further burden on existing resources.

## 5. Conclusions

In summary, POS is quick, simple, feasible, cost-effective, acceptable, and improves the detection rate for CCHD and significantly reduces mortality. POS meets the criteria for universal screening and has been adopted into routine postnatal practice in many high-income countries. POS also identifies a significant number of babies with potentially life-threatening non-cardiac conditions, particularly in the first 24 h of life, which is an important additional benefit.

The most effective screening algorithm is still a subject of debate, but it is important to remember that whichever algorithm is used, all are superior to existing screening methods. Perhaps a balance needs to be struck between the detection of serious illness and limiting false-positive results, and local circumstances may have an influence in this respect. More data from larger populations may help to refine the screening algorithm further, and PI (perhaps with input from ML) may play an additional role. Awareness that current POS is not a perfect test and babies with CCHDs may still be missed is also very important; health care workers and parents need to be aware of the limitations of the test. Further investigation of alternative strategies to identify challenging defects such as LHODs should be encouraged.

## Data Availability

No new data were created or analyzed in this study. Data sharing is not applicable to this article.

## Abbreviations

CCHD—critical congenital heart defect, POS—pulse oximetry screening, AAO—aortic arch obstruction, CoA—coarctation of the aorta, IAA—interrupted aortic arch, PI—perfusion index.
